# Investigating Nurses' Competencies for Development of “Internet + Nursing Service”: A Cross‐Sectional Study

**DOI:** 10.1002/nop2.70275

**Published:** 2025-07-24

**Authors:** Jialing Chen, Qi Zhang, Pedro Fong, Lirong Meng

**Affiliations:** ^1^ Faculty of Health Sciences and Sports Macao Polytechnic University Macao People's Republic of China; ^2^ The Second Affiliated Hospital of Soochow University Soochow China

**Keywords:** digital health, innovation, internet + nursing service, nursing informatics, telehealth

## Abstract

**Aim:**

To test whether nursing informatics competency (NIC) and innovation ability predict nurses' willingness for “Internet + nursing service” in China's Greater Bay Area, and examine the role of innovation ability in this relationship.

**Design:**

Cross‐sectional study.

**Review Methods:**

Recruited registered nurses via quota sampling in China's Greater Bay Area. Participants completed validated scales assessing NIC, Innovation Behaviour, and Willingness for Internet + nursing service, testing a hypothesised mediation model where innovation ability links informatics competency to willingness.

**Data Sources:**

Data were collected from 544 registered nurses between January and March 2024.

**Results:**

Nurses reported moderate levels of NIC, innovation ability, and willingness for Internet + nursing service. Higher informatics competency was associated with greater innovation ability and willingness. Furthermore, innovation ability appeared as a pathway linking informatics competency to willingness.

**Conclusion:**

Stronger NIC and innovation ability may boost nurses' readiness for Internet‐enabled care in the Greater Bay Area.

**Implications for the Profession and/or Patient Care:**

Targeted informatics and innovation training can enhance nurses' readiness for these digital services.

**Impact:**

Addressing the need for enhanced digital nursing (‘Internet + nursing service’) for the aging population in China's Greater Bay Area, this study found moderate nurse informatics competency, innovation ability, and willingness to engage in this service. Higher competency and innovation were linked to greater willingness, with informatics competency influencing willingness directly and through innovation ability. These findings impact nursing practice, education, and policy in the region, informing strategies to improve digital service readiness.

**Reporting Method:**

This study adheres to the Strengthening the Reporting of Observational Studies in Epidemiology (STROBE) guidelines.

**Patient or Public Contribution:**

No patients or members of the public were involved in study design, conduct, reporting, or dissemination.

## 1 Introduction

The Guangdong‐Hong Kong‐Macao Greater Bay Area (GBA) is a prominent bay area urban agglomeration in China, encompassing nine Pearl River Delta cities in Guangdong and the two special administrative regions of Hong Kong and Macao. Supported significantly by national policies, the GBA plays a crucial role in China's economic development (Lee and Lin 2020) and is comparable in growth trajectory to renowned bay areas like San Francisco, New York, and Tokyo (Hui et al. 2020).

However, this rapid development coincides with significant healthcare challenges, primarily the dual pressures of a rapidly aging population and strains on the nursing workforce (Yang et al. 2023). The demographic shift is stark: estimates from 2023 indicated that approximately 22% of Hong Kong's population was aged 65 or older, classifying it as a ‘super‐aged society’, while the nine mainland GBA cities collectively had around 7% aged 65+ (PwC China 2024), though this proportion is rising. Projections suggest this trend will accelerate dramatically, with Hong Kong's elderly population expected to reach 36% by 2046 (PwC China 2024). This profound demographic shift fuels an escalating demand for complex elderly care services, particularly long‐term care and management for the high prevalence of chronic diseases within this age group, services which often require significant nursing resources.

This rising demand for elderly care confronts challenges in service capacity and workforce availability. Demand significantly exceeds supply, particularly in Hong Kong and Macao, exacerbated by factors like limited land and healthcare professional shortages (Lam 2022). Consequently, individuals face long waiting times for essential community and residential care services; for example, average waits in Hong Kong stretched from 6 to 24 months in mid‐2022 (Social Welfare Department of Hong Kong 2022). Concurrently, the nursing workforce faces persistent pressures, evidenced by high attrition rates in Hong Kong public hospitals (9.5% in 2023–2024) (Pinkstone 2024) and the implementation of cross‐border nurse exchange programs launched in 2023–2024 to alleviate shortages, particularly in areas like elderly care (Wu 2024). Recent GBA‐specific policy initiatives, such as expanding access to cross‐border elderly care facilities and extending Hong Kong's Elderly Health Care Voucher scheme to mainland GBA cities in 2024 (Greater Bay Healthcare 2024; Secretary for Health 2025), further underscores that managing the growing elderly care needs amidst resource constraints, including nursing staff, is a critical regional priority.

To address these pressing issues, the Chinese government established the “Internet + nursing service” initiative, aiming to improve healthcare service efficiency and promote social equity by leveraging internet technology within healthcare (Zhang et al. 2020). This model enables registered nurses to provide essential healthcare services—such as remote consultation, health education, chronic disease management, and rehabilitation guidance (Shen et al. 2021)—directly to patients in their homes, especially benefiting elderly individuals or those with restricted mobility.

The successful implementation and widespread adoption of this innovative service model are vital for addressing the GBA's healthcare challenges, but this hinges significantly on the participation and willingness of the nursing workforce. Therefore, understanding the factors that influence nurses' readiness to engage in this new technology‐enabled mode of practice is essential for developing effective strategies to promote its adoption and maximise its potential benefits within the GBA. This study aimed to investigate key professional competencies—specifically Nursing Informatics Competency (NIC) and Nursing Innovation Ability (NIA) – and their relationship with nurses' willingness to participate in the “Internet + nursing service” in the GBA.

## 2 Background

“Internet + nursing service” typically involves medical institutions utilising information technology to coordinate registered nurses providing in‐home care, often facilitated through online application platforms, followed by offline service delivery. Officially piloted in six provincial‐level regions in 2019, including Guangdong, the initiative was expanded nationally in 2021 (Yang et al. 2021). Despite its expansion, the model is still considered to be in its developmental stages in China.

Previous research, predominantly conducted in various regions of mainland China, has explored nurses' willingness to participate in “Internet + nursing service,” yielding inconsistent findings (Xu and Xie 2022). Reported willingness rates have varied considerably, for example, from 20.43% in one Guangdong study (Xu and Xie 2022) to 60.94% in a Jiangxi study (Xu 2020). These discrepancies may be attributable to regional differences in culture, healthcare resources, demographics, or nursing professionals' competencies (Li, Krumholz, et al. 2020). Notably, research specifically focusing on the unique context of the GBA, which includes the distinct healthcare systems and cultures of Hong Kong and Macao (Kong et al. 2015) has been lacking.

Effective adoption of technology‐enabled nursing services like “Internet + nursing service” likely depends on specific professional competencies. NIC, broadly defined as the integration of nursing knowledge, skills, and attitudes with computer and information science capabilities to manage data and deliver care (Kleib and Nagle 2018a; Staggers et al. 2001), is fundamental for using the digital tools inherent in such services. NIA, described as the capacity to proactively explore, devise, and implement novel techniques or technologies to enhance healthcare and patient care (Yan, Yang, et al. 2018), may foster receptiveness to new service models. Prior studies have suggested a positive association between NIA and willingness for telenursing (Ling 2021) and a positive correlation between NIC and NIA (Yan, Sun, et al. 2018), implying that informatics skills may support innovation. Furthermore, a systematic review conducted by Bei et al. (2023) on distance care reveals that information and communication technology (ICT) barriers can negatively affect the provision and adoption of telecare. This finding highlights the importance of informatics competency for nurses to adopt information technology‐related nursing work. Also, the mechanism through which NIA influences nurses' willingness to participate in the service remains unclear. We believe that understanding these knowledge gaps could enhance the implementation of the “Internet + nursing service” more effectively.

This study's conceptual framework is guided by the Theory of Planned Behaviour (TPB) (Ajzen 2020). TPB posits that behavioural intention (willingness, in this context) is determined by attitudes towards the behaviour, subjective norms, and perceived behavioural control (Ajzen 2020)—constructs assessed by the willingness scale used herein (Li et al. 2022). Recognising that TPB can be effectively extended by incorporating additional relevant variables (Ajzen 2015), our framework includes NIC and NIA as key individual characteristic variables hypothesised to influence nurses' willingness to participate in ‘Internet + nursing service’.

Specifically, building on prior correlational findings (Ling 2021; Yan, Sun, et al. 2018), the framework proposed and tested a mediating pathway where NIC positively influences NIA, and both NIC and NIA, in turn, positively influence willingness. This addressed several key knowledge gaps. Prior research had not explicitly examined the direct effect of NIC on willingness for this specific service model. Furthermore, the mechanism through which NIC and NIA might jointly influence willingness, particularly the hypothesised mediating role of NIA, requires empirical investigation. The distinct cultural and healthcare environments of the GBA also necessitated region‐specific research (Kong et al. 2015; Li, Krumholz, et al. 2020).

Therefore, this study sought to investigate the current levels of NIC, NIA, and willingness among nurses in the GBA, explore the correlations among these factors, test the hypothesised mediating role of NIA, and identify significant predictors of willingness within this specific population and context.

## 3 Methods

### 3.1 Design

This report has adhered to the Strengthening the Reporting of Observational Studies in Epidemiology (STROBE) guidelines. This is a cross‐sectional study using a quota sampling method to recruit clinical nurses in hospitals in the GBA.

### 3.2 Sample Size and Power

The sample size for participants (*N*) was determined using the following equation:

$$N=\frac{z^{2}\sigma^{2}}{\delta^{2}}$$

*z* equals 1.65, indicating the statistical confidence level at a test level of *α* = 0.1. The overall standard deviation (*σ*) of nursing willingness to engage in the service was 0.65; this value was based on a previous study (Qiao et al. 2020). An allowable error of 0.05 was used as the value of 𝛿. To account for potential errors and loss of recoverable samples and invalid questionnaires, the sample size was moderately increased by 10%, resulting in a final sample size of 506 cases.

### 3.3 Sampling and Recruitment

Due to the voluntary nature of nurse participation in this survey, random sampling of participating nurses was not feasible. A quota sampling method was then employed. All the registered nurses in the GBA were stratified based on the 2020 Guangdong Provincial Health Statistical Yearbook, the 2021 Hong Kong Nursing Council statistics, and the 2021 Macau Statistics and Census Bureau statistics. This involved dividing the participants into four strata based on the number of registered nurses in each city. Cities with a large number of registered nurses, such as Guangzhou and Shenzhen, were grouped into two strata. Cities with a small number of registered nurses, such as Zhuhai, Jiangmen, Zhaoqing, Huizhou, Zhongshan, Dongguan, and Foshan, were grouped into one stratum. Hong Kong and Macao were combined into one stratum due to their small size and similar historical and cultural backgrounds. After the stratification, the total number of registered nurses at each stratum was calculated based on the statistical data of the registered nurses in each city. The number of participants was calculated to be 139 for Guangzhou, 77 for Shenzhen, 204 for other cities in the Pearl River Delta, and 86 for the Hong Kong and Macao regions.

### 3.4 Inclusion and Exclusion Criteria

The inclusion criteria of the participants were as follows: holding a nurse qualification certificate in the corresponding area, being a registered nurse, having worked in nursing for at least 1 year at the surveyed hospital, and voluntarily agreeing to participate in the study by signing the informed consent form. All nurses who were not working at the surveyed hospitals, such as advanced nurses and practice nurses, were excluded from this study.

### 3.5 Instrument With Validity and Reliability

This study employed four distinct questionnaires to collect data on four elements: NIC, innovation ability, nurses' willingness to engage in “Internet + nursing service,” and demographic information. The questionnaires were originally completed in Chinese, and the English versions provided in Appendices S1–S4 were created solely for the readers of this article.

The “Assessment Scale for Nursing Informatics Competency” (Appendix S1) was used to measure NIC. This scale was originally developed in English by Kleib and Nagle (2018b) and was later modified and translated into Chinese by Chen (2021) The scale consists of four dimensions with 21 items, which assess basic information and communication technology (ICT) competency (2 items), information management competency (7 items), ethics and law (6 items), and nursing information practice competency (6 items). All items were evaluated using a 4‐point Likert scale, with scores ranging from “not consistent” (1 point) to “completely consistent” (4 points). The total score is between 21 and 84 points, with higher scores indicating stronger nursing information competency. This specific scale (the Chinese version of C‐NICAS) was selected because it is based on established competency frameworks (Staggers, TIGER), was recently validated in Chinese clinical nurses demonstrating good psychometric properties (Chen 2021), and provides a concise yet comprehensive assessment across key informatics domains relevant to practice. Chen (2021) reported high internal consistency (Cronbach's *α* = 0.950) and confirmed its factor structure, supporting its validity for measuring NIC in this study's context.

The innovation ability was measured by the “Innovation Behaviour Scale” (Appendix S2) developed by Lukes and Stephan (2017), which was modified and translated into Chinese by Huang et al. (2021) The scale consists of 5 dimensions with 20 items, including idea generation and search (6 items), planning communication and implementation (5 items), acquisition of human resources (3 items), overcoming obstacles (3 items), and clinical application (3 items). All items are rated on a 5‐point Likert scale, ranging from “strongly agree” (5 points) to “strongly disagree” (1 point). The total score ranges from 20 to 100 points. This study divided the scores into four levels: “20–40 points”, “40–60 points”, “60–80 points”, and “80–100 points”, with higher scores indicating a higher level of NIA. This Scale was chosen for its detailed assessment of multiple stages of innovative behaviour (from idea generation to application), its established cross‐cultural psychometric properties, and its successful validation specifically among Chinese nurses (Huang et al. 2021). Huang et al. (2021) reported good overall reliability (Cronbach's *α* = 0.923) and construct validity for the Chinese version, making it suitable for assessing NIA in this sample.

The “Scale of Nurses' Participation Willingness in ‘Internet + nursing service’” (Li et al. 2022) (Appendix S3) was used to measure the nurses' willingness to engage in “Internet + nursing service”. It consists of three subscales with a total of 17 items. The subscales include the attitude towards participation (5 items), subjective norms (5 items), and perceived behavioural control (7 items). All items were rated on a 5‐point Likert scale, with 1 indicating “completely disagree,” 2 indicating “disagree,” 3 indicating “neutral,” 4 indicating “agree,” and 5 indicating “completely agree.” Items number 10, 12, and 13 were reverse scored. The total score ranges from 17 to 85 points, with higher scores indicating a greater willingness to engage in “Internet + nursing services”. This instrument was selected as it was specifically developed to measure nurses' willingness towards “Internet + nursing service” within the Chinese context, its development was explicitly guided by the TPB, which underpins this study's framework, and it demonstrated good reliability and validity in the original development study (Li et al. 2022). Li et al. (2022) reported strong overall reliability (Cronbach's *α* = 0.927) and evidence of criterion‐related validity, supporting its use for measuring willingness in this study.

Finally, a self‐designed questionnaire was used to collect participants' demographic and professional background information (Appendix S4). This included details such as age, gender, years of work experience, job title, highest education level, marital status, specialist nurse status, monthly income, self‐perceived health condition, and familiarity with “Internet + nursing services”. This information was deemed necessary to accurately describe the study sample's characteristics, provide essential context for interpreting the primary findings, and allow for the exploration of potential relationships between these background variables and nurses' willingness to engage in “Internet + nursing service”.

### 3.6 Data Collection

The data were collected using the online platform known as “Wen Juan Xing” (Changsha Ranxing Information Technology Co. Ltd., Hunan, China) which offers services that are comparable to those provided by popular survey tools such as SurveyMonkey and CloudResearch. The platform enables users to design and administer questionnaires online. The platform has over 2.6 million members, and their personal information is verified. This platform has yielded meaningful results in numerous academic studies.

All the data collectors were either nurses or nursing students who underwent training on how to accurately administer the electronic questionnaires via online video workshops. A data collection manual was prepared for the training. The data collectors were responsible for distributing the electronic questionnaires to the designated hospitals in the form of QR codes. They also collected electronic copies of the participants' informed consent and guided questionnaire completion at the hospital. Alternatively, participants were allowed to complete the questionnaire at their convenience and were advised to contact the data collectors for any queries or concerns. Participants were restricted to one response per “Wen Juan Xing” ID, and completion of all questions was mandatory for successful submission.

### 3.7 Ethics

This study was approved by the ethical committee of Macao Polytechnic University (Reference No: FCSD/MSN‐036/2022, Approved Date: September 22, 2022). All the participants were explained the study's purpose, content, and the time required to complete the questionnaires before providing their informed consent. The data collection process did not cause any harm to the participants. Participant privacy and confidentiality were maintained throughout the study. Data were collected anonymously via the ‘Wen Juan Xing’ online platform, ensuring that no personal identifiers (such as names or specific hospital affiliations beyond the region) were linked to the responses. Completed questionnaires were stored securely in password‐protected files accessible only to the research team, and it was assured that the content would be used solely for this research study. All results are reported in an aggregated format to prevent the identification of individual participants.

### 3.8 Statistical Analysis

The data from Wen Juan Xing was downloaded and imported into SPSS (version 24.0). Independent sample *t*‐tests and one‐way ANOVA were employed to analyse differences in the willingness to engage in “Internet + nursing service” among nurses with different demographic characteristics. Pearson correlation analysis was conducted to explore the correlation between three variables: nurses' informatics competency, innovation ability, and willingness to engage in “Internet + nursing service.” Multiple linear regression analysis with the ENTER method was used to study the factors influencing nurses' willingness to engage in “Internet + nursing service.” To test the hypothesised mediating effect of NIA between NIC and willingness, mediation analysis was performed using the PROCESS macro (Model 4) for SPSS (Hayes 2018), employing bias‐corrected bootstrapping with 5000 resamples and 95% confidence intervals.

## 4 Results

### 4.1 Reliability and Validity of Instruments

A pilot study (*N* = 201 valid responses) was conducted prior to the full‐scale survey. Internal consistency for the main scales was good (Cronbach's *α* > 0.8), although the Willingness subscales of Subjective Norms and Perceived Behavioural Control showed lower but acceptable reliability (*α* = 0.687 and 0.541, respectively) (Table 1). Exploratory factor analysis (EFA) supported the structural validity of the Chinese versions of the Assessment Scale for NIC, Innovation Behaviour Scale (NIA), and Scale of Nurses' Participation Willingness in “Internet + nursing service” (Willingness) used in this study. KMO values were > 0.9, and Bartlett's tests were significant (*p* < 0.001) for all scales. EFA extracted the expected number of factors consistent with the original scale designs, with cumulative variance explained exceeding 50% and all item factor loadings > 0.400 (Table 2).

**TABLE 1:** Reliability of the “Assessment Scale for Nursing Informatics Competency”, “Innovation Behaviour Scale” and “Scale of Nurses' Participation Willingness in ‘Internet + nursing services’”.

Scales	No. of items	Cronbach's *α*
Assessment Scale for Nursing Informatics Competency	21	0.971
D1 Basic information and communication technology competency	2	0.883
D2 Information management competency	7	0.935
D3 Ethics and law	6	0.927
D4 Nursing information practice competency	6	0.947
Innovation Behaviour Scale	20	0.957
D1 Idea generation and search	6	0.896
D2 Planning communication and implementation	5	0.871
D3 Acquisition of human resources	3	0.841
D4 overcoming obstacles	3	0.878
Scale of Nurses' Participation Willingness in “Internet + nursing services”	17	0.870
S1 Attitude towards participation	5	0.885
S2 Subjective norms	5	0.687
S3 Perceived behavioural control	7	0.541

**TABLE 2:** Exploratory factor analysis of the “Assessment Scale for Nursing Informatics Competency”, “Innovation Behaviour Scale” and “Scale of Nurses' Participation Willingness in ‘Internet + nursing services’”.

Scales	KMO values	Bartlett *p*	Factors	Eigen values	Variance explained %
Assessment Scale for Nursing Informatics Competency	0.952	< 0.001	1	7.175	78.723
			2	5.077	
			3	3.126	
			4	1.154	
Innovation Behaviour Scale	0.944	< 0.001	1	5.373	77.060
			2	3.838	
			3	3.161	
			4	2.138	
			5	1.902	
Scale of Nurses' Participation Willingness in “Internet + nursing services”	0.915	< 0.001	1	6.009	64.608
			2	2.996	
			3	1.979	

### 4.2 Participant Characteristics

Including the 229 questionnaires from the pilot study, a total of 588 questionnaires were distributed, out of which 544 questionnaires were collected and considered valid, resulting in a completion rate of 92.52%. Among them, there were 124 from Hong Kong and Macau, 139 from Guangzhou, 77 from Shenzhen, and 204 from other cities in the Pearl River Delta region.

The majority of participants were unmarried (58.8%), female (89.9%), aged 26–35 (54.2%), with 1–5 years of experience (57.7%), bachelor's degrees (79.2%), earning 5000–10,000 RMB (Chinese official currency)/month (37.7%), with good health (57.4%), and had heard of “Internet + nursing service” (65.8%), while 24.3% were not familiar with it. In terms of job titles, the majority of participants held the positions of nurse/primary nurse or below (44.1%), with only 18.6% being specialised nurses. Their willingness to engage in this service varied significantly with job title, marital status, specialist status, income, health perception, and familiarity with the service (*p* < 0.05), but not with age, gender, years of experience, or education level (*p* > 0.05) (Table 3).

**TABLE 3:** Demographic information and scores on willingness to engage in “Internet + nursing services” among 544 participants.

	*N* (%)	Scores (mean ± SD)	*t*/*F*	*p*
Age (years)				
≤ 25	165 (30.3)	3.50 ± 0.42	0.77	0.512
26~35	295 (54.2)	3.55 ± 0.45		
36~45	69 (12.7)	3.56 ± 0.55		
> 45	15 (2.8)	3.44 ± 0.47		
Gender				
Female	489 (89.9)	3.54 ± 0.45	0.83	0.408
Male	55 (10.1)	3.48 ± 0.49		
Years of nursing experience (years)				
1–5	314 (57.7)	3.52 ± 0.43	0.636	0.599
6–10	119 (21.9)	3.54 ± 0.46		
11–15	50 (9.2)	3.51 ± 0.46		
> 15	61 (11.2)	3.60 ± 0.55		
Job Title (Mainland China/Hong Kong and Macao)				
Nurse/level 1 nurse or below^a^	240 (44.1)	3.45 ± 0.43	6.08	< 0.001
Nurse practitioner/specialist^b^	221 (40.6)	3.56 ± 0.46		
Charge nurse/nursing leader	77 (14.2)	3.69 ± 0.45		
Chief nurse or above/supervisor	6 (1.1)	3.73 ± 0.49		
Highest education level				
Diploma/associate degree	59 (10.8)	3.56 ± 0.47	0.58	0.561
Bachelor's degree	432 (79.4)	3.52 ± 0.45		
Master's degree or above	53 (9.7)	3.58 ± 0.51		
Marital status				
Unmarried	320 (58.8)	3.48 ± 0.44	5.40	0.005
Married	219 (40.1)	3.61 ± 0.45		
Divorced	6 (1.1)	3.58 ± 0.65		
Specialist nurse^c^				
Yes	101 (18.6)	3.66 ± 0.47	3.14	0.002
No	443 (81.4)	3.60 ± 0.45		
Monthly income (*RMB*)^d^				
< 5000	63 (11.6)	3.45 ± 0.40	9.23	< 0.001
5000–10,000	205 (37.7)	3.54 ± 0.46		
10,000–20,000	158 (29.0)	3.66 ± 0.43		
> 20,000	118 (21.7)	3.39 ± 0.46		
Perspective in own health				
Good	312 (57.4)	3.62 ± 0.45	13.32	< 0.001
Average	210 (38.6)	3.42 ± 0.42		
Poor	22 (4.0)	3.41 ± 0.57		
Familiarity with “Internet + nursing services”				
Very well known (attended training)	54 (9.9)	3.85 ± 0.47	42.69	< 0.001
Fairly well known (heard of/looked up)	358 (65.8)	3.58 ± 0.42		
Not known	142 (24.3)	3.27 ± 0.42		

^a^ This category combines the baseline registered nurse ranks surveyed, corresponding to ‘Nurse’ in Mainland China and ‘Level 1 Nurse’ in Hong Kong/Macao systems.

^b^ ‘Nurse practitioner/specialist’ refers to a job title category within the surveyed GBA nursing structures.

^c^ Indicates whether the participant holds a formal specialist nurse certification or recognised designation.

^d^ RMB stands for Renminbi, the official currency of China. The approximate US Dollar (USD) equivalent based on the average exchange rate in 2024 is 1 USD≈7.17 RMB.

### 4.3 Descriptive Statistics of Key Variables

Table 4 displays the scores for NIC, innovation ability, and willingness to engage in “Internet + nursing service” for the 544 participants. The average NIC score was 2.80 ± 0.62 (total score 58.75 ± 13.09). The ethics and law dimension scored highest (2.90 ± 0.66), while information management competency scored lowest (2.67 ± 0.69). The average NIA score was 3.91 ± 0.54 (total score 78.29 ± 10.82). The acquisition of the human resources dimension was highest (4.05 ± 0.56), and clinical application was lowest (3.76 ± 0.71). The average Willingness score was 3.53 ± 0.45 (total score 60.06 ± 7.73). The perceived behavioural control subscale scored highest (22.90 ± 3.02), while the subjective norms subscale scored lowest (17.75 ± 2.70). Nurses in the Pearl River Delta had significantly higher scores for NIC, NIA, and Willingness compared to those in Hong Kong and Macao (*p* < 0.05, Table 5). NIC and NIA were positively correlated (*r* = 0.509, *p* < 0.01). Additionally, all dimensions of NIC and NIA showed positive correlations with the total Willingness score (correlation values ranging from 0.236 to 0.603, Table 6).

**TABLE 4:** Scores of nursing informatics competency, innovation ability and nurses' willingness to engage in “Internet + nursing services” among the 544 participants.

	No. of items	Total scores	Average scores
Nursing informatics competency	21	58.75 ± 13.09	2.80 ± 0.62
Basic ICT	2	5.58 ± 1.59	2.79 ± 0.80
Information management	7	18.71 ± 4.81	2.67 ± 0.69
Ethics and law	6	17.39 ± 3.97	2.90 ± 0.66
Nursing information practice	6	17.07 ± 4.12	2.85 ± 0.69
Innovation ability	20	78.29 ± 10.82	3.91 ± 0.54
Idea generation and search	6	24.11 ± 3.35	4.02 ± 0.56
Planning communication, and implementation	5	19.24 ± 3.23	3.85 ± 0.65
Acquisition of human resources	3	12.15 ± 1.69	4.05 ± 0.56
Overcoming obstacles	3	11.49 ± 1.98	3.83 ± 0.66
Clinical application	3	11.29 ± 2.12	3.76 ± 0.71
Nurses' willingness to engage in “Internet + nursing services”	17	60.06 ± 7.73	3.53 ± 0.45
Attitude towards participation	5	19.40 ± 3.03	3.88 ± 0.61
Subjective norms	5	17.75 ± 2.70	3.55 ± 0.54
Perceived behavioural control	7	22.90 ± 3.02	3.27 ± 0.43

**TABLE 5:** Scores of nursing informatics competency, innovation ability and nurses' willingness to engage in “Internet + nursing services” among the 544 participants in the Pearl River Delta region, Hong Kong and Macao.

Scales	Pearl River Delta (*N* = 420)	Hong Kong and Macao (*N* = 124)	*t*	*p*
Nursing informatics competency				
Total score	59.38 ± 13.36	56.62 ± 11.93	2.069	0.039
Average score of all questions	2.83 ± 0.64	2.70 ± 0.57		
Innovation ability				
Total score	79.57 ± 10.73	73.95 ± 9.97	5.202	< 0.001
Average score of all questions	3.98 ± 0.54	3.70 ± 0.50		
Nurses' willingness to engage in “Internet + nursing services”				
Total score	61.00 ± 7.60	56.85 ± 7.31	5.384	< 0.001
Average score of all questions	3.59 ± 0.45	3.34 ± 0.43		

**TABLE 6:** Values of Pearson correlation (*r*) between the scores of each dimension of nursing informatics competency and innovation ability with the total score of willingness to engage in “Internet + nursing services”.

	Correlation (*r*)
Nursing informatics competency	0.423**
Basic ICT	0.236**
Information management	0.383**
Ethics and law	0.381**
Nursing information practice	0.439**
Innovation ability	0.603**
Idea generation and search	0.534**
Planning communication, and implementation	0.592**
Acquisition of human resources	0.425**
Overcoming obstacles	0.517**
Clinical application	0.507**

** Indicates *p* < 0.01.

### 4.4 Predictors of Willingness: Multiple Linear Regression Analysis

The analysis revealed a significant positive correlation between the dependent variable, willingness to engage in the “Internet + nursing service”, and the independent variables: familiarity with the service, NIC, and innovation ability (Table 7). These three variables were used to construct a regression model that accounted for 40.8% of the variance (*R* ^2^ = 0.408). The F value for the regression equation was 40.913, and the *p*‐value was < 0.01, indicating that the regression model was statistically significant. The resulting regression equation was:

$$\begin{array}{c} Y_{\text{willingness to engage}}=1.353+0.1X_{\text{familiarity}}\\ \;+0.105X_{\text{informatics competency}}\\ \;+0.386X_{\text{innovation competency}} \end{array}$$

**TABLE 7:** Multiple linear regression analysis of the nurses' willingness to engage in the “Internet + nursing services” and other variables.

Variables	*B*	SE	*β*	*t*	*p*
Constant	1.353	0.135	—	10.009	< 0.001
Job title	0.025	0.024	0.041	1.058	0.291
Marital status					
Unmarried	0.017	0.036	0.018	0.475	0.635
Married	−0.013	0.147	−0.003	−0091	0.928
Specialist nurse	0.035	0.041	0.030	0.849	0.396
Monthly income	0.009	0.017	0.019	0.550	0.582
Perspective on own health	0.043	0.028	0.055	1.559	0.120
Familiarity with “Internet + nursing services”	0.100	0.310	0.125	3.247	0.001
Nursing informatics competency	0.105	0.029	0.144	3.669	< 0.001
Innovation ability	0.386	0.035	0.459	11.129	< 0.001

*Note:* *R* ^2^ = 0.408, adjusted *R* ^2^ = 0.398, *F* = 40.913, *p* < 0.01.

### 4.5 Mediation Analysis

Based on the results of the correlation analysis in this study, it is known that the three variables under investigation exhibit positive correlations with each other. To further investigate the interrelationships among these three variables, and incorporating the results from the multiple linear regression analysis, a mediation effect model was constructed. NIC was designated as the independent variable (X), NIA as the mediating variable (M), and willingness to engage as the dependent variable (Y). Given that the degree of understanding significantly influences the willingness to engage, it was included as a control variable. The Bootstrap method was employed to test the mediating effect of NIA on the relationship between NIC and willingness to engage in “Internet + nursing services”.

Using MODEL 4 within the PROCESS programme, the mediation effect analysis was conducted with 5000 bootstrap samples and a 95% confidence interval. A confidence interval not containing zero indicates a significant mediating effect. The results showed that the confidence intervals for the effects in the mediation model did not include zero, and all *p*‐values were < 0.001, confirming the significance of each effect (see in Table 8). This indicates that the mediation model is valid and represents a partial mediation. The magnitude of the mediating effect was 0.194, accounting for 63.0% of the total effect value (0.308). Details can be found in Table 9.

**TABLE 8:** Mediation model test for innovation ability.

Result variable	Predictor variable	*β*	SE	*t*	Boot 95% CI	*R* ^2^	*F*
Mediator variable	Constant	2.679	0.092	29.141***	(2.499, 2.860)	0.259	189.428***
	Independent variable	0.442	0.032	13.763***	(0.379, 0.505)		
Dependent variable	Constant	1.492	0.113	13.186***	(1.270, 1.715)	0.382	166.967***
	Independent variable	0.114	0.029	3.992***	(0.058, 0.171)		
	Mediator variable	0.44	0.033	13.319***	(0.375, 0.504)		

*** Indicates *p* < 0.001.

**TABLE 9:** Results of mediation effect test.

Effects	Effect values	Boot SE	Boot 95% CI
Total	0.308	0.028	(0.253–0.364)
Direct	0.114	0.029	(0.058–0.170)
Indirect	0.194	0.022	(0.153–0.240)

Abbreviations: Boot, bootstrap; CI, confidence interval; SE, standard error.

**FIGURE 1:**
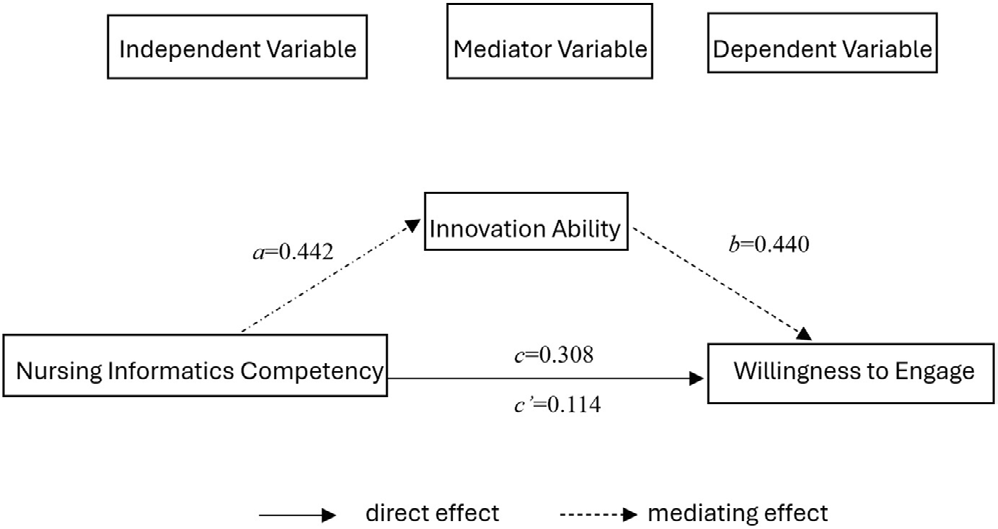
Mediation effect model diagram. The product *a* × *b* represents the mediation effect, *c'* denotes the direct effect, and *c* signifies the total effect.

In summary, the analysis outlines the mediating effect of NIA on the relationship between NIC and nurses' willingness to engage in “Internet + nursing services”, as illustrated in Figure 1.

## 5 Discussion

This study aimed to investigate NIC, NIA, and willingness to participate in “Internet + nursing service” among nurses in the Guangdong‐Hong Kong‐Macao Greater Bay Area (GBA), exploring the relationships between these factors and the mediating role of NIA.

### 5.1 Psychometric Properties of Study Instruments

Before discussing the main findings, it is pertinent to analyse the reliability and validity of the scales employed. The pilot study indicated good internal consistency for the main scales measuring NIC and Innovation Behaviour. The Scale of Nurses' Participation Willingness in “Internet + nursing services” also showed acceptable overall reliability, although the subscales for Subjective Norms and Perceived Behavioural Control demonstrated lower internal consistency. While these lower values warrant cautious interpretation of findings related specifically to those sub‐dimensions, the overall scale reliability supports its use. EFA further supported the structural validity of the Chinese versions of the NIC, NIA, and Willingness scales used. Factor analysis suitability indicators (KMO and Bartlett's test) were strong. The EFA extracted the expected number of factors consistent with the original scale designs, with substantial cumulative variance explained and strong item factor loadings, confirming the construct validity of the instruments within this study's sample.

### 5.2 Participant Characteristics

The demographic profile of the participants—primarily female (89.9%), aged 26–35 (54.2%), with 1–5 years of experience (57.7%) and holding junior titles (Nurse/level 1 nurse or below, 44.1%)—aligns with the general nursing workforce structure in the region (Yang and Li 2021). However, the predominance of nurses with bachelor's degrees (79.4%) contrasts with the broader Chinese context, where specialised (diploma‐level) education has historically been more common (Yang and Li 2021), likely reflecting higher educational requirements in the surveyed GBA hospitals. Consistent with previous studies (Li, Gou, et al. 2020; Qiao et al. 2020), a majority of nurses reported low familiarity with “Internet + nursing services” (65.8% ‘fairly well known’, 24.3% ‘not known’), potentially due to the service's early developmental stage in the GBA and insufficient promotion and popularisation.

### 5.3 Nursing Informatics Competency, Innovation Ability and Nurses' Willingness to Engage

This study found that the average score for NIC was (Mean = 2.80, SD = 0.62), indicating a moderate level of competency. This contrasts with a previous study (Chai 2021) conducted in other regions of mainland China, which reported a lower average score (Mean = 2.11, SD = 0.89). The difference in scores could be due to the higher proportion (> 50%) of participants with a bachelor's or above degree in this study, as previous research demonstrated a positive association between informatics competency and higher educational levels (Kleib and Nagle 2018b).

The participants from the GBA obtained an average score of (Mean = 3.91, SD = 0.54) in innovation ability, which is higher than the results obtained from other regions in mainland China (Zhao 2022), with an average score of (Mean = 3.17, SD = 1.01). This difference could be attributed to the higher proportion of participants with a bachelor's or higher degree in this study, as individuals with higher educational levels may possess greater innovation ability (Xie and Ji 2016). Additionally, significant resources invested by China to promote innovation in the Pearl River Delta region may have cultivated an environment conducive to enhancing nurses' innovation ability. Of all the dimensions of the NIA, clinical application had the lowest score (Mean = 3.76, SD = 0.71). In Zhao's (2022) study, the dimension with the lowest score was the acquisition of human resources. The reason for this difference may be that the GBA, being one of China's most open and economically vibrant regions with advanced innovation resources, allows nurses easier access to new information, fostering idea generation. However, the low score in clinical application might be linked to the high population density and severe nurse shortage in the GBA, leaving limited time for clinical innovation application.

This study found that the average score for nurses' willingness to engage in “Internet + nursing service” was (Mean = 3.53, SD = 0.45), indicating a moderate willingness to participate. This result is consistent with a previous study (Qiao et al. 2020) conducted in other regions of mainland China, which reported a similar score (Mean = 3.56, SD = 0.65). The dimension with the highest average score in this study was the attitude towards participation (Mean = 3.88, SD = 0.61), whereas perceived behavioural control had the lowest score (Mean = 3.27, SD = 0.43). This contrasts with the results of Qiao et al. (2020), possibly due to the advanced innovative atmosphere of the GBA fostering positive attitudes. However, the relatively low promotion of “Internet + nursing service” in Hong Kong and Macao may lead to a lack of understanding among nurses about the service requirements and development, potentially hindering their ability to judge their capacity or institutional support, thus contributing to the lower score in perceived behavioural control.

Furthermore, this study found that nurses in the Pearl River Delta region have stronger nursing information and innovation competencies, and are more willing to engage in “Internet + nursing service” than those in the Hong Kong and Macao regions (all *p* < 0.05). This may be attributed to the denser population and relatively limited resources in the Pearl River Delta, leading to a more competitive nursing job market that necessitates continuous professional development. Moreover, evaluation criteria emphasising research abilities, including NIC and NIA, are often applied in Mainland China's hospitals (Liao et al. 2023). In contrast, universities in Hong Kong and Macao generally place a higher emphasis on clinical practice education (Zhong et al. 2021).

### 5.4 Factors Influencing Willingness

Our findings indicate that higher job titles, specialist nurse status, better self‐perceived health, and greater familiarity with the service are associated with higher willingness to participate (*p* < 0.05, Table 3). This aligns with previous research (Han et al. 2020; Qiao et al. 2020). Higher‐ranking nurses often possess more experience and skills, fostering confidence in embracing new service models (Liao et al. 2023). Specialist nurses may see these services as opportunities to utilise their expertise and find value, potentially compensating for limited specialised roles in traditional settings. Better health likely provides the energy needed to engage with new initiatives, while greater familiarity enhances confidence and understanding of the service. Married nurses also demonstrated significantly higher willingness (*p* < 0.05, Table 3). While the reasons require further exploration, this finding might be linked to potential increased family financial responsibilities, possibly making married individuals more receptive to supplemental income opportunities offered by participating in ‘Internet + nursing service’. However, this interpretation remains speculative.

The relationship with income was nuanced: willingness increased with income up to 20,000 RMB/month, but dropped significantly for those earning above this threshold (*p* < 0.001, Table 3). Higher earners (up to 20 k) often have stronger skills and may be eager to try novel services. The lowest willingness in the > 20,000 RMB group likely reflects that this cohort predominantly comprised Hong Kong and Macao nurses (108 out of 118), who have less familiarity with the service due to its limited promotion in those regions.

Consistent with prior research (Guo et al. 2024), NIC was positively correlated with NIA (*r* = 0.509, *p* < 0.01) (see Table 6). Stronger information skills likely enable nurses to access and utilise information effectively, fostering problem‐solving and innovative ideas. Both NIC and NIA were positively correlated with willingness to participate (*p* < 0.01, Table 6). Higher informatics competency equips nurses to use the required technologies, increasing comfort and willingness, aligning with findings that competent nurses are more inclined towards remote healthcare (Ma et al. 2021). Greater innovation ability fosters exploration of new models (Ling 2021) and is linked to higher self‐evaluation and work enthusiasm (Wu et al. 2014), translating into greater willingness for novel services.

Multiple linear regression confirmed familiarity, NIC, and NIA as significant positive predictors of willingness, explaining 40.8% of the variance (*R* ^2^ = 0.408, *p* < 0.001, Table 7). This underscores that promoting the service and enhancing these competencies are key strategies to foster participation.

Crucially, the mediation analysis revealed that NIA partially mediated the relationship between NIC and Willingness (indirect effect = 0.194, accounting for 63.0% of the total effect). This suggests that while informatics skills directly boost willingness (perhaps through technical confidence), they also enhance innovation ability (e.g., by facilitating information access for creative solutions), which in turn further increases willingness to engage in new services like “Internet + nursing service”.

### 5.5 Strengths and Limitations

This study has several strengths. It is one of the first to investigate NIC, NIA, and willingness for “Internet + nursing service” specifically within the unique GBA context, encompassing diverse healthcare systems. The use of established, validated questionnaires enhances the reliability of the findings. Furthermore, the exploration of NIA's mediating role provides novel insights into the mechanism linking informatics competency and willingness.

However, certain limitations must be acknowledged. First, the use of quota sampling, while pragmatic, may limit the generalizability of the findings to the entire GBA nursing population compared to probability sampling methods. Second, the lower reliability coefficients observed for the Subjective Norms and Perceived Behavioural Control subscales of the Willingness measure warrant caution when interpreting results specific to these dimensions. Third, the significant differences observed between the Pearl River Delta and Hong Kong/Macao regions suggest caution in applying findings uniformly across the entire GBA, highlighting the need for region‐specific strategies. Finally, this study focused solely on nurses' perspectives; incorporating patient and family views is crucial for a holistic understanding of “Internet + nursing service” adoption.

### 5.6 Implications for Nursing and International Relevance

The findings hold significant implications for nursing practice, education, and policy, particularly relevant in the context of global healthcare trends. The study highlights that enhancing nurses' NIC and NIA is crucial for fostering engagement in technology‐enabled care models like “Internet + nursing service”, which are increasingly vital worldwide.

For nursing practice, targeted training programmes should focus not only on basic ICT skills but specifically on information management and the clinical application of innovation, areas where competencies were relatively lower. Globally, as healthcare systems grapple with ageing populations and workforce shortages, equipping nurses with strong informatics skills is essential for managing complex patient data, ensuring patient safety through technology, and improving workflow efficiency (American Nurses Association 2023; Kleib et al. 2021). Initiatives to increase familiarity with the service model itself are also necessary.

For nursing education, curricula should systematically integrate informatics and innovation competencies. This aligns with international recommendations to embed nursing informatics training early and throughout nursing education to prepare graduates for digitally transformed healthcare environments (Forman et al. 2020; Kleib et al. 2021). Educational institutions in the GBA could benchmark against international competency frameworks like TIGER to ensure graduates are adequately prepared. Emphasis should be placed on practical application and ethical considerations related to health data management.

For policy, the findings support the need for policies that actively promote and standardise “Internet + nursing service” across the GBA, addressing the observed regional disparities. This includes developing clear guidelines, ensuring adequate institutional support and resources, and potentially exploring medical reimbursement models. Learning from international experiences, establishing supportive regulatory frameworks and addressing financial constraints are key factors in facilitating the successful adoption and scaling of digital health solutions for aging populations (Iyamu and Gómez‐Ramírez 2022).

The challenges faced by the GBA—a rapidly aging population and strained nursing resources—mirror global trends driving the adoption of telehealth and digital nursing solutions. The “Internet + nursing service” model, while specific to China, represents a national effort to leverage technology for home‐based care, reflecting a worldwide movement towards using digital tools, smart devices, and telehealth platforms to enhance care access, efficiency, and independence for older adults (Tan et al. 2025). This study's confirmation of the pivotal roles of NIC and NIA resonates with the international recognition that these competencies are fundamental for nurses to effectively navigate and leverage technology in modern healthcare settings. By demonstrating the interplay between these competencies and willingness in the unique GBA setting, this research contributes valuable regional evidence to the global discourse on preparing the nursing workforce for a digitally‐driven future of healthcare delivery, particularly in serving aging populations.

## 6 Conclusion

This study investigated if NIC and innovation ability (NIA) predict nurses' willingness for “Internet + nursing service” in China's Greater Bay Area (GBA) and examined NIA's mediating role. Findings confirm that strong nursing information competency and innovation ability are positively associated with the willingness to engage in the services, with innovation ability partially mediating the relationship. Given these results, enhancing NIC and NIA through targeted training, like informatics education and innovation competitions, is crucial. Such efforts, combined with promoting service familiarity, can increase engagement, helping the GBA address healthcare challenges like population aging via this service model. Future studies could explore the perspectives of patients and families in the GBA regarding their willingness to use such services.

## Author Contributions

**Jialing Chen:** conceptualisation, investigation, formal analysis, writing – original draft. **Qi Zhang:** investigation, formal analysis, writing – review and editing. **Pedro Fong:** conceptualisation, methodology, writing – original draft, writing – review and editing. **Lirong Meng:** conceptualisation, methodology, writing – review and editing, project administration.

## Disclosure

Statistics declaration: The authors have checked to make sure that our submission conforms as applicable to the Journal's statistical guidelines. Lirong Meng, a member of the author's team, served as the statistician. The authors affirm that the methods used in the data analyses are suitably applied to the data within this study design and context, and the statistical findings have been implemented and interpreted correctly. The authors agree to take responsibility for ensuring that the choice of statistical approach is appropriate and is conducted and interpreted correctly as a condition to submit to the Journal.

## Conflicts of Interest

The authors declare no conflicts of interest.

## Supporting information


Appendices S1–S4

## Data Availability

The data that support the findings of this study are available from the corresponding author upon reasonable request.

## References

[nop270275-bib-0001] Ajzen, I. 2015. “The Theory of Planned Behaviour Is Alive and Well, and Not Ready to Retire: A Commentary on Sniehotta, Presseau, and Araújo‐Soares.” Health Psychology Review 9, no. 2: 131–137. 10.1080/17437199.2014.883474. 26209198

[nop270275-bib-0002] Ajzen, I. 2020. “The Theory of Planned Behavior: Frequently Asked Questions.” Human Behavior and Emerging Technologies 2, no. 4: 314–324. 10.1002/hbe2.195.

[nop270275-bib-0003] American Nurses Association . 2023. “What Is Nursing Informatics and Why Is It So Important?” Accessed May 5, 2025. https://www.nursingworld.org/content‐hub/resources/nursing‐resources/nursing‐informatics/.

[nop270275-bib-0004] Bei, E. , V. Morrison , M. Zarzycki , and N. Vilchinsky . 2023. “Barriers, Facilitators, and Motives to Provide Distance Care, and the Consequences for Distance Caregivers: A Mixed‐Methods Systematic Review.” Social Science & Medicine 317: 115782. 10.1016/j.socscimed.2022.115782. 36801750

[nop270275-bib-0005] Chai, X. 2021. “A Study on the Influence of Clinical Nurses' Nursing Information Competence on Their Perception of Patient Safety Culture.” Chinese General Practice Nursing 19, no. 2: 281–284. 10.12404/j.issn.1674-4748.2021.02.040.

[nop270275-bib-0006] Chen, X. 2021. The Study of Revising and Verifying the Nursing Practice Information Competency Assessment Scale. Master's thesis, Qingdao University.

[nop270275-bib-0007] Forman, T. M. , D. A. Armor , and A. S. Miller . 2020. “A Review of Clinical Informatics Competencies in Nursing to Inform Best Practices in Education and Practice.” Nursing Education Perspectives 41, no. 1: E3–E7. 10.1097/01.NEP.0000000000000584. 31860501

[nop270275-bib-0008] Greater Bay Healthcare . 2024. “GBA Healthcare Updates: Key Policy Changes in 2024.” https://www.google.com/search?q=https://www.greaterbayhealthcare.info/post/gba‐healthcare‐updates‐key‐policy‐changes‐in‐2024.

[nop270275-bib-0009] Guo, J. , J. Liu , C. Liu , Y. Wang , X. Xu , and Y. Chen . 2024. “Nursing Informatics Competency and Its Associated Factors Among Palliative Care Nurses: An Online Survey in Mainland China.” BMC Nursing 23, no. 1: 157. 10.1186/s12912-024-01803-5. 38443955 PMC10913251

[nop270275-bib-0010] Han, M. , M. Zhao , X. Zhang , L. Zhang , and M. Wang . 2020. “Investigation on Nurses' Knowledge of and Willingness to Participate in Internet Plus Nursing Service.” Journal of Nursing Science 35, no. 4: 53–56.

[nop270275-bib-0011] Hayes, A. F. 2018. Introduction to Mediation, Moderation, and Conditional Process Analysis: A Regression‐Based Approach. 2nd ed. Guilford Press.

[nop270275-bib-0012] Huang, L. , Y. Chen , J. Wang , and Y. Zhang . 2021. “Reliability and Validity Testing of Chinese Version Innovative Behavior Scale (IBI) in Chinese Nurses.” Journal of Nursing Science 36, no. 7: 81–84.

[nop270275-bib-0013] Hui, E. C. M. , X. Li , T. Chen , and W. Lang . 2020. “Deciphering the Spatial Structure of China's Megacity Region: A New Bay Area—The Guangdong‐Hong Kong‐Macao Greater Bay Area in the Making.” Cities 105: 102168. 10.1016/j.cities.2018.10.011.

[nop270275-bib-0014] Iyamu, I. , and O. Gómez‐Ramírez . 2022. “Challenges in the Development of Digital Public Health Interventions and Mapped Solutions: Findings From a Scoping Review.” Digital Health 8: 1–17. 10.1177/20552076221102255. PMC9152201 35656283

[nop270275-bib-0015] Kleib, M. , A. Chauvette , K. Furlong , L. Nagle , L. Slater , and R. McCloskey . 2021. “Approaches for Defining and Assessing Nursing Informatics Competencies: A Scoping Review.” JBI Evidence Synthesis 19, no. 4: 794–841. 10.11124/JBIES-20-00100. 33625068

[nop270275-bib-0016] Kleib, M. , and L. Nagle . 2018a. “Development of the Canadian Nurse Informatics Competency Assessment Scale and Evaluation of Alberta's Registered Nurses' Self‐Perceived Informatics Competencies.” CIN: Computers, Informatics, Nursing 36, no. 7: 350–358. 10.1097/CIN.0000000000000435. 29668498

[nop270275-bib-0017] Kleib, M. , and L. Nagle . 2018b. “Psychometric Properties of the Canadian Nurse Informatics Competency Assessment Scale.” CIN: Computers, Informatics, Nursing 36, no. 7: 359–365. 10.1097/CIN.0000000000000436. 29634497

[nop270275-bib-0018] Kong, X. , Y. Yang , J. Gao , et al. 2015. “Overview of the Health Care System in Hong Kong and Its Referential Significance to Mainland China.” Journal of the Chinese Medical Association 78, no. 10: 569–573. 10.1016/j.jcma.2015.02.006. 26144022

[nop270275-bib-0019] Lam, G. 2022. “Shortage of Nurses in Hong Kong: The Challenges Ahead.” Asian Education and Development Studies 12, no. 1: 89–102. 10.1108/AEDS-08-2021-0179.

[nop270275-bib-0020] Lee, I. , and R. F.‐Y. Lin . 2020. “Economic Complexity of the City Cluster in Guangdong–Hong Kong–Macao Greater Bay Area, China.” Sustainability 12, no. 14: 5639. 10.3390/su12145639.

[nop270275-bib-0021] Li, J. , J. Gou , Q. Zhou , Z. Hu , and X. Zheng . 2020. “Investigation and Analysis on Willingness of Nurse to Use Internet + Nursing Services in Non‐Pilot Provinces.” Chinese Journal of Nursing 55, no. 12: 1825–1830.

[nop270275-bib-0022] Li, S. , J. Yu , and B. Xu . 2022. “Compilation and Reliability and Validity Test of Participation Intention of Nurses on Internet Plus Nursing Service Scale.” Chinese Journal of Modern Nursing 28, no. 2: 205–209.

[nop270275-bib-0023] Li, X. , H. M. Krumholz , W. Yip , et al. 2020. “Quality of Primary Health Care in China: Challenges and Recommendations.” Lancet 395, no. 10239: 1802–1812. 10.1016/S0140-6736(20)30122-7. 32505251 PMC7272159

[nop270275-bib-0024] Liao, S. , J. Lai , X. Gong , W. Huang , and H. Song . 2023. “A Study on the Current Situation of Nursing Human Resources Allocation in Guangdong Province From 2014 to 2020.” Chongqing Medicine 52, no. 4: 623–628.

[nop270275-bib-0025] Ling, W. 2021. Research on Influencing Factors of Nurses' Intention to Use Telenursing Service. Master's thesis, Southern Medical University.

[nop270275-bib-0026] Lukes, M. , and U. Stephan . 2017. “Measuring Employee Innovation: A Review of Existing Scales and the Development of the Innovative Behavior and Innovation Support Inventories Across Cultures.” International Journal of Entrepreneurial Behavior and Research 23, no. 1: 136–158. 10.1108/IJEBR-11-2015-0262.

[nop270275-bib-0027] Ma, N. , B. Wang , and D. Wang . 2021. “Status Quo of Nurses' Remote Health Care Readiness and Its Influencing Factors.” Chinese Nursing Research 35, no. 20: 3588–3593.

[nop270275-bib-0028] Pinkstone, J. 2024. Hong Kong Public Hospital Nurses Resigned at Record Rate Last Year, Hospital Authority Figures Show. Hong Kong Free Press. https://www.google.com/search?q=https://hongkongfp.com/2024/03/06/hong‐kong‐public‐hospital‐nurses‐resigned‐at‐record‐rate‐last‐year‐hospital‐authority‐figures‐show/.

[nop270275-bib-0029] PwC China . 2024. “Forging a Collective Future: PwC GBA Healthcare Report 2024.” https://www.google.com/search?q=https://www.pwccn.com/en/industries/healthcare/publications/gba‐healthcare‐report‐2024.html.

[nop270275-bib-0030] Qiao, H. , W. Gao , N. Luo , et al. 2020. “Influencing Factors of Nurses' Willingness to Engage in ‘Internet + Nursing Service’.” Nursing Journal of Chinese People's Liberation Army 37, no. 9: 30–33.

[nop270275-bib-0031] Secretary for Health . 2025. LCQ11: Elderly Health Care Voucher Scheme. Press Releases, HKSAR Government. https://www.google.com/search?q=https://www.info.gov.hk/gia/general/202501/15/P2025011500190.htm.

[nop270275-bib-0032] Shen, W. , Y. Qin , Z. Shi , K. Tang , X. Chen , and Z. Li . 2021. “Analysis of the Current Situation and Influencing Factors of Nurses' Demand for Internet Plus Nursing Service at Different Levels in China.” Chinese Journal of Hospital Administration 37, no. 4: 326–331.

[nop270275-bib-0033] Social Welfare Department of Hong Kong . 2022. “Community Support Services for the Elderly.” Accessed April 27, 2023. https://www.google.com/search?q=https://www.swd.gov.hk/en/index/site_pubsvc/page_elderly/sub_csselderly/id_introduction/.

[nop270275-bib-0034] Staggers, N. , C. A. Gassert , and C. Curran . 2001. “Informatics Competencies for Nurses at Four Levels of Practice.” Journal of Nursing Education 40, no. 7: 303–316. 10.3928/0148-4834-20011001-05. 11596683

[nop270275-bib-0035] Tan, S. H. , Y. Y. Yap , S. K. Tan , and C. K. Wong . 2025. “Determinants of Telehealth Adoption Among Older Adults: Cross‐Sectional Survey Study.” JMIR Aging 8: e60936. 10.2196/60936. 40126531 PMC11976177

[nop270275-bib-0036] Wu, A. , X. Tong , S. Liang , and S. Jiang . 2014. “Relationship Between Core Self‐Evaluations and Innovative Behaviors in Nurses.” Journal of Nursing Science 29, no. 12: 39–41.

[nop270275-bib-0037] Wu, C. 2024. “Guangdong Nurses Start Work in Hong Kong Under Greater Bay Area Exchange Scheme.” *South China Morning Post*. https://www.google.com/search?q=https://www.scmp.com/news/hong‐kong/health‐environment/article/3258589/guangdong‐nurses‐start‐work‐hong‐kong‐under‐greater‐bay‐area‐exchange‐scheme.

[nop270275-bib-0038] Xie, X. , and P. Ji . 2016. “Factors Influencing Innovative Behavior of Operating Room Nurses and Improvement Measures.” Nei Mongol Journal of Traditional Chinese Medicine 35, no. 3: 116–117. 10.16040/j.cnki.cn15-1101.2016.03.134.

[nop270275-bib-0039] Xu, L. , and Y. Xie . 2022. “Influencing Factors of Nurses' Willingness to Engage in ‘Internet Plus Nursing Service’.” Modern Hospital 22, no. 2: 259–262.

[nop270275-bib-0040] Xu, X. 2020. Nanchang Third Class A Hospital Clinical Nurses on Internet: Investigation and Analysis on the Willingness of Nursing Service. Master's thesis, Nanchang University. Chinese Master's Theses Database.

[nop270275-bib-0041] Yan, J. , L. Sun , J. Yan , and Q. Zhou . 2018. “Correlation Between Information Literacy and Innovative Behavior Among Nurses.” Journal of Nursing Science 33, no. 23: 69–70.

[nop270275-bib-0042] Yan, J. , J. Yang , Y. Jiang , M. Zhao , and H. Wang . 2018. “Development of Nurse Innovation Ability Scale and Its Reliability and Validity Testing.” Chinese Journal of Nursing 53, no. 10: 1213–1217.

[nop270275-bib-0043] Yang, C. , C. Y. Ma , J. Wang , and Y. Zhou . 2023. “Cross‐Border Ageing in China's Greater Bay Area in the Digital Age: A Comparative Study of Mobile Application Adoption by Hong Kong Older Migrants and Local Older Adults in Shenzhen.” Transactions in Planning and Urban Research 2, no. 1: 149–166. 10.1177/27541223221150653.

[nop270275-bib-0044] Yang, F. , H. Shu , and X. Zhang . 2021. “Understanding “Internet Plus Healthcare” in China: Policy Text Analysis.” Journal of Medical Internet Research 23, no. 7: e23779. 10.2196/23779. 34309581 PMC8367124

[nop270275-bib-0045] Yang, Z. , and N. Li . 2021. “Research on the Equity of the Nursing Human Resource Allocation in China, 2013–2017.” Modern Preventive Medicine 48, no. 5: 858–861.

[nop270275-bib-0046] Zhang, Q. , M. Li , and Y. Wu . 2020. “Smart Home for Elderly Care: Development and Challenges in China.” BMC Geriatrics 20: 318. 10.1186/s12877-020-01737-y. 32883224 PMC7650273

[nop270275-bib-0047] Zhao, H. 2022. “A Study on the Current Situation and Influencing Factors of Innovative Behavior of Emergency Department Nursing Staff.” Modern Nurse 29, no. 2: 118–121.

[nop270275-bib-0048] Zhong, X. , X. Xu , M. Li , J. Li , and W. Huang . 2021. “Current Status of Practice Teaching of Nursing Undergraduates in Guangdong‐Hong Kong‐Macao Greater Bay Area and Its Enlightenment.” Journal of Nursing (China) 28, no. 20: 16–21.

