# Supplementary material for: Investigating Nurses' Competencies for Development of “Internet + Nursing Service”: A Cross‐Sectional Study
Source: Nurs Open. 2025 Jul 24;12(7):e70275. doi: 10.1002/nop2.70275 (PMC12289534; doi:10.1002/nop2.70275)
Supplement: Supplementary file 1 — Appendices S1–S4 [file NOP2-12-e70275-s001.docx]

**Appendix S1.** Assessment Scale for Nursing Informatics Competency

1. I am proficient in using information technology equipment such as computers, USB devices, printers, projectors, and other related devices.

□ Highly compliant

□ Moderately compliant

□ Somewhat compliant

□ Not at all compliant

1. I am proficient in using information and communication application such as email, Microsoft Word, Microsoft Excel, Microsoft PowerPoint, internet browsers, and other related applications.

□ Highly compliant

□ Moderately compliant

□ Somewhat compliant

□ Not at all compliant

1. I am able to search for and critically evaluate online scholarly literature and information resources.

□ Highly compliant

□ Moderately compliant

□ Somewhat compliant

□ Not at all compliant

1. I am capable to record, interpret, and analyse relevant nursing and patient data using standardized nursing language, such as nursing diagnostics.

□ Highly compliant

□ Moderately compliant

□ Somewhat compliant

□ Not at all compliant

1. I am capable of helping patients and their families navigate, review, and assess online information from various sources, including social media sites, smartphone apps, and health-related organizational documents.

□ Highly compliant

□ Moderately compliant

□ Somewhat compliant

□ Not at all compliant

1. I have the ability to proficiently collect, enter, and retrieve data from both paper-based and electronic nursing records.

□ Highly compliant

□ Moderately compliant

□ Somewhat compliant

□ Not at all compliant

1. I understand the importance of standardized information in interoperable electronic medical records. (Interoperability refers to the ability to exchange and share data across different platforms or systems.)

□ Highly compliant

□ Moderately compliant

□ Somewhat compliant

□ Not at all compliant

1. I understand the importance of standardizing nursing data to reflect nursing practice and improve nursing knowledge.

□ Highly compliant

□ Moderately compliant

□ Somewhat compliant

□ Not at all compliant

1. I possess the ability to critically evaluate data and information from multiple reliable sources, including experts, clinical applications, databases, practice guidelines, and related websites, and use it to guide care delivery.

□ Highly compliant

□ Moderately compliant

□ Somewhat compliant

□ Not at all compliant

1. I adhere to laws and regulations, ethical principles, and hospital systems to protect the privacy and security of patient information.

□ Highly compliant

□ Moderately compliant

□ Somewhat compliant

□ Not at all compliant

1. I advocate for the use of existing and innovative information and communication technologies (ICTs) in healthcare systems.

□ Highly compliant

□ Moderately compliant

□ Somewhat compliant

□ Not at all compliant

1. I am able to identify and report system errors, functional defects, and equipment failures in the medical information system in a timely manner according to hospital rules and procedures.

□ Highly compliant

□ Moderately compliant

□ Somewhat compliant

□ Not at all compliant

1. I have demonstrated the ability to maintain effective nursing practices and ensure patient safety during periods of system outages.

□ Highly compliant

□ Moderately compliant

□ Somewhat compliant

□ Not at all compliant

1. I apply professional judgment when using existing technologies and systems.

□ Highly compliant

□ Moderately compliant

□ Somewhat compliant

□ Not at all compliant

1. I believe that nurses should be involved in the design, selection, implementation, and evaluation of information and communication equipment and systems as they play an essential role in the process.

□ Highly compliant

□ Moderately compliant

□ Somewhat compliant

□ Not at all compliant

1. I am proficient in using various information and communication technologies, such as PDAs, HIS systems, electronic medical records, various monitoring devices, and wearable devices, to provide care.

□ Highly compliant

□ Moderately compliant

□ Somewhat compliant

□ Not at all compliant

1. I use decision support tools, such as clinical alerts, clinical pathways, and practice guidelines, to assist in professional judgment.

□ Highly compliant

□ Moderately compliant

□ Somewhat compliant

□ Not at all compliant

1. I use information and communication technology as a means of supporting the caregiver-patient relationship.

□ Highly compliant

□ Moderately compliant

□ Somewhat compliant

□ Not at all compliant

1. I am able to describe the various modules contained in the hospital information system.

□ Highly compliant

□ Moderately compliant

□ Somewhat compliant

□ Not at all compliant

1. I am able to describe the various electronic records used in clinical nursing practice.

□ Highly compliant

□ Moderately compliant

□ Somewhat compliant

□ Not at all compliant

1. I can explain how informatics can improve the quality of care and the healthcare environment.

□ Highly compliant

□ Moderately compliant

□ Somewhat compliant

□ Not at all compliant

**Appendix S2.** Innovation Behaviour Scale

1. I enjoy trying out new ways of working in nursing.

□ Strongly agree □ Agree □ Neither agree nor disagree □ Disagree □ Strongly disagree

1. I prefer jobs that involve innovative ideas, such as scientific research and innovative inventions.

□ Strongly agree □ Agree □ Neither agree nor disagree □ Disagree □ Strongly disagree

1. When facing problems in clinical work, I attempt to find new solutions.

□ Strongly agree □ Agree □ Neither agree nor disagree □ Disagree □ Strongly disagree

1. I seek new ideas from colleagues or nursing professionals.

□ Strongly agree □ Agree □ Neither agree nor disagree □ Disagree □ Strongly disagree

1. I am also interested in exploring external areas to apply new ideas to clinical work.

□ Strongly agree □ Agree □ Neither agree nor disagree □ Disagree □ Strongly disagree

1. I strive to achieve the best solution by seeking new ideas from others.

□ Strongly agree □ Agree □ Neither agree nor disagree □ Disagree □ Strongly disagree

1. When I have a new idea, I attempt to obtain support from the head nurse.

□ Strongly agree □ Agree □ Neither agree nor disagree □ Disagree □ Strongly disagree

1. I aim to present the positive aspects of new ideas to my colleagues.

□ Strongly agree □ Agree □ Neither agree nor disagree □ Disagree □ Strongly disagree

1. When I have a new idea, I involve individuals who can collaborate.

□ Strongly agree □ Agree □ Neither agree nor disagree □ Disagree □ Strongly disagree

1. I make appropriate plans and timelines to implement new ideas.

□ Strongly agree □ Agree □ Neither agree nor disagree □ Disagree □ Strongly disagree

1. I seek and acquire the necessary funding to implement new ideas.

□ Strongly agree □ Agree □ Neither agree nor disagree □ Disagree □ Strongly disagree

1. If problems arise during the implementation process, I seek help from individuals who can solve them.

□ Strongly agree □ Agree □ Neither agree nor disagree □ Disagree □ Strongly disagree

1. When implementing an idea, I involve key decision-makers.

□ Strongly agree □ Agree □ Neither agree nor disagree □ Disagree □ Strongly disagree

1. When implementing an idea, I seek individuals who have the ability to make it work.

□ Strongly agree □ Agree □ Neither agree nor disagree □ Disagree □ Strongly disagree

1. I persist in overcoming obstacles when implementing an idea.

□ Strongly agree □ Agree □ Neither agree nor disagree □ Disagree □ Strongly disagree

1. If I fail to reach my goal, I usually do not give up easily.

□ Strongly agree □ Agree □ Neither agree nor disagree □ Disagree □ Strongly disagree

1. During the implementation process, even if things do not go well, I persevere.

□ Strongly agree □ Agree □ Neither agree nor disagree □ Disagree □ Strongly disagree

1. I frequently succeed in implementing my ideas at work and putting them into practice.

□ Strongly agree □ Agree □ Neither agree nor disagree □ Disagree □ Strongly disagree

1. Many of the ideas that I conceive are utilized within our organization.

□ Strongly agree □ Agree □ Neither agree nor disagree □ Disagree □ Strongly disagree

1. No matter where I work, I strive to improve things there.

□ Strongly agree □ Agree □ Neither agree nor disagree □ Disagree □ Strongly disagree

**Appendix S3.** Scale of Nurses' Participation Willingness in "Internet + Nursing Services"

1. I believe that taking part in "Internet + nursing services" has more advantages than disadvantages and can enhance the value of nursing professionals.

□ Strongly agree □ Agree □ Neither agree nor disagree □ Disagree □ Strongly disagree

1. I believe that taking part in "Internet + Nursing Services" can enable me to serve more patients in need and bring me happiness.

□ Strongly agree □ Agree □ Neither agree nor disagree □ Disagree □ Strongly disagree

1. I believe that I can gain more benefits by participating in "Internet + Nursing Services."

□ Strongly agree □ Agree □ Neither agree nor disagree □ Disagree □ Strongly disagree

1. I believe that "Internet + nursing services" is a new concept, a path to self-realisation.

□ Strongly agree □ Agree □ Neither agree nor disagree □ Disagree □ Strongly disagree

1. I believe that participating in "Internet + Nursing Services" can shape my personal image and expand my influence.

□ Strongly agree □ Agree □ Neither agree nor disagree □ Disagree □ Strongly disagree

1. I will participate in "Internet + Nursing Services" due to the support and guidance of the leaders in my hospital.

□ Strongly agree □ Agree □ Neither agree nor disagree □ Disagree □ Strongly disagree

1. I will participate in "Internet + Nursing Services" due to the recommendation of my colleagues at my hospital.

□ Strongly agree □ Agree □ Neither agree nor disagree □ Disagree □ Strongly disagree

1. I will participate in "Internet + Nursing Services" due to the views of experts in related fields.

□ Strongly agree □ Agree □ Neither agree nor disagree □ Disagree □ Strongly disagree

1. I will participate in "Internet + Nursing Services" based on the advice of friends and family.

□ Strongly agree □ Agree □ Neither agree nor disagree □ Disagree □ Strongly disagree

1. I am concerned that participating in "Internet + Nursing Services" will take up a significant amount of my time and increase my burden.

□ Strongly agree □ Agree □ Neither agree nor disagree □ Disagree □ Strongly disagree

1. I am confident that I can provide excellent service to patients independently through "Internet + Nursing Services."

□ Strongly agree □ Agree □ Neither agree nor disagree □ Disagree □ Strongly disagree

1. I am aware of the potential risks associated with participating in "Internet + Nursing Services."

□ Strongly agree □ Agree □ Neither agree nor disagree □ Disagree □ Strongly disagree

1. I am familiar with the relevant policies of government departments regarding the development of "Internet + nursing services," and I will consider participating in this service.

□ Strongly agree □ Agree □ Neither agree nor disagree □ Disagree □ Strongly disagree

1. I believe that the change in physical location when participating in "Internet + Nursing Services" may affect the quality of nursing care provided.

□ Strongly agree □ Agree □ Neither agree nor disagree □ Disagree □ Strongly disagree

1. I believe that the risks associated with participating in "Internet + nursing services" outweigh the benefits obtained.

□ Strongly agree □ Agree □ Neither agree nor disagree □ Disagree □ Strongly disagree

1. I have received basic technical training on how to participate in "Internet + Nursing Services."

□ Strongly agree □ Agree □ Neither agree nor disagree □ Disagree □ Strongly disagree

1. I find it easy to understand the registration, certification, audit, login, and other necessary steps before participating in "Internet + nursing services."

□ Strongly agree □ Agree □ Neither agree nor disagree □ Disagree □ Strongly disagree

**Appendix S4.** Personal Background Information Questionnaire

1. Age: □ ≤25 years old

□ 26~35 years old

□ 36~45 years old

□ >45 years old

1. Gender: □ female

□ male

1. Years of nursing experience: □ 1─5 years

□ 6─10 years

□ 11─15 years

□ >15 years

1. Job Title:

*For mainland China -* □ nurse

□ nurse practitioner

□ charge nurse

□ deputy chief nurse or above

*For Hong Kong and Macao* - □ level 1 nurse or below

□ specialist/advanced specialist

□ deputy nursing leader/nursing leader

□ practice supervisor

1. Highest education level: □ below undergraduate level

□ bachelor's degree

□ master's degree or above

1. Marital status: □ unmarried

□ married

□ divorced

□ widowed

1. Specialist nurse: □ Yes

□ No

1. Average monthly income (RMB): □ <5,000

□ 5,000─10,000

□ 10,000─20,000

□ >20,000

1. Your perspective on your own health: □ good

□ average

□ poor

1. Did you know about "Internet + Nursing Services"?

□ very well-known (attended training)

□ fairly well known (heard of/looked up)

□ not known
